# Facilitators and barriers to Tuberculosis case notification among private health facilities in Kampala Capital City, Uganda

**DOI:** 10.1371/journal.pone.0315402

**Published:** 2024-12-19

**Authors:** Veronica Kembabazi, Julius Ssentongo, Elizeus Rutebemberwa

**Affiliations:** 1 Department of Health Policy, Planning and Management, Makerere University School of Public Health, Kampala, Uganda; 2 ResilientAfrica Network, Makerere University School of Public Health, Kampala, Uganda; University of Zimbabwe Faculty of Medicine: University of Zimbabwe College of Health Sciences, ZIMBABWE

## Abstract

**Introduction:**

Private Health Facilities (PHFs), Uganda’s main healthcare providers, are indispensable stakeholders in the national tuberculosis (TB) program’s efforts to improve TB case notification and combat the epidemic. However, notification rates remain relatively low in PHFs compared to public providers. In this study, we sought to assess facilitators and barriers to TB case notification among private facilities in Kampala Capital City.

**Methods:**

We conducted a cross-sectional study utilizing a mixed-methods approach to assess facilitators and barriers to TB notification in Kampala Capital City between March and July 2022. For the quantitative strand of the study, we interviewed the TB focal persons at 224 PHFs using a structured questionnaire and for the qualitative, we conducted 14 key informant and in-depth interviews with Ministry of Health-Uganda staff, and TB focal persons at the Kampala Division administration level and at the PHFs. The quantitative analysis involved Modified Poisson regression and the qualitative analysis was carried out using thematic analysis to identify the facilitators and barriers to TB case notification.

**Results:**

Of the 224 PHFs surveyed, the majority, 39.3%(88), were facilities in Nakawa division and 55.4% (124) of the respondents were male, with a mean age of 32.6 years (SD = 8.6). We found that the prevalence of TB case notification was significantly lower for facilities in Kawempe (PR 0.16; 95%CI 0.05,0.47) and Nakawa (PR 0.39, 95%CI 0.21,0.73). Notification was lower among facilities that had no guide for TB screening and diagnosis (PR 0.50; 95%CI 0.25,0.97) and among those facilities where training of other health workers at the facility in TB diagnosis was unknown (PR 0.35; 95%CI 0.13,0.93). Qualitative data showed that the main facilitators of TB case notification were: regular engagements between the NTLP and private health providers and, provision of materials and support to conduct case finding, while the main barriers included TB stigma, lack of resources such as TB diagnostic facilities.

**Conclusions:**

PHFs in Kampala Capital City are receptive to programmatic TB case notification. However, they need regular supervision and engagement activities to ensure that they have updated knowledge, equipment and funding support to carry out TB case notification according to policy.

## Introduction

TB is a leading cause of death, accounting for 1.3 million deaths globally [1] and over 30% of these deaths occur in Africa [2]. Moreover, the number of new cases seems to be increasing: for example, an estimated 10.6 million cases were reported in 2022, 9.9 million in 2020, and 6.4 million in 2017 [3, 4]. In Uganda, approximately 240 people fall ill with TB or lose their lives to disease daily [5].

TB case notification is a process involving screening, diagnosis, referral and reporting TB cases to the National TB programme. Robust TB case notification is crucial for assessing the country’s burden of TB and planning and evaluating the effectiveness of TB programs. Given the significance of TB case notification, Uganda has made it mandatory for health facilities to report all presumptive TB cases. However, despite this policy, Uganda still has significant gaps in the notification of TB cases. The Uganda National TB prevalence survey of 2014/15 showed that the prevalence of TB was more than twice the reported TB cases suggesting gaps in detection and reporting [6]. TB case notification rates in the PHFs are estimated to be as low as 20% [7, 8]. This is a significant challenge to improving overall notification rates, as PHFs are the primary healthcare providers, especially in urban areas [9–11].

Delays in TB case notification tend to lead to the development of drug-resistant TB [12]. Past studies found that TB diagnosis delays were also significantly associated with attendance of a PHF [13]. Private Health Providers (PHPs) therefore play a key role in reducing delays in case finding as well as provision of TB care [14, 15]. It is essential that these private facilities are linked to the public sector and where necessary, provided with guidance, equipment and infrastructure to provide appropriate TB services.

The National TB programs have gradually been engaging these PHF to link them to TB program support. Since 2019, the National TB and Leprosy Control Programme (NTLP), Uganda, has increased efforts to improve TB diagnosis and notification rates through procuring and installing various diagnostic tools such as GeneXpert machines, Chest X-ray machines to diagnose and record TB cases in facilities, some of which are available for use by private healthcare facilities [16].

In spite of the widespread knowledge that PHF are lagging in TB case notification and that they are critical in urban areas to improving overall notification rates, there is limited understanding of the barriers or facilitators to Tuberculosis notification by PHPs in Uganda. In this study, we sought to assess facilitators and barriers to TB case notification among private health facilities in Kampala Capital City to guide efforts towards improving TB case notification among private health facilities in the area.

## Materials and methods

### Study area

The study was conducted in Kampala, the Capital city of Uganda. In mid-2020, it had a population of 1,680,600 as estimated by UBOS [17]. Kampala was selected because PHPs make up 98% of all health providers in Kampala due to the high level of private health care attendance and a high TB burden. Uganda had an estimated TB incidence rate of 200 per 100,000 [18] with Kampala’s incident TB cases at an estimated 8,380 people [16] in 2020.

There were 1395 private clinics, HC IIs and HC IIIs registered by MoH as of 2020 in all the five (5) divisions of Kampala. Lower-level health facilities (Clinics to Health centre IIIs) serve populations of up to 20,000 while higher-level facilities serve bigger populations between 100,000 to 10,000,000. These facilities may be Private, Faith-based, Community-based or Non-governmental Organization owned facilities and the majority of these are Private for Profit (PFP). The Private for Profit mainly consist of lower-level facilities that offer preventive, promotive, outpatient curative health services, outreach, laboratory and maternity services. Whereas higher-level facilities are mostly Private not for Profit and Government facilities that offer, in addition to lower-level facility health services, in-patient, radiology, specialist services, surgery and research. This study was conducted among selected private facilities across selected divisions of Kampala.

### Study population

This study was conducted in Kampala district; in private (PFP&PNFP) clinics, HCIIs and HCIIIs that are reporting in DHIS2. All health facilities are expected to report data on TB screening, TB cases diagnosed and TB treatment at their facility. Hospitals and Health Centre IVs were not included because, based on DHIS2 data, TB case notification is especially a problem in lower-level facilities like Clinics, Health Centre IIs and some Health Centre IIIs and conducting the study among them would provide more relevant data for our study. The target population was the above groups of private facilities in Kampala City registered in DHIS2. Although laboratories may do bacteriological diagnosis of TB, they were left out of this study since they do not report into DHIS2.

#### Inclusion and exclusion criteria

Selected private health Centre IIs, IIIs and clinics in the selected divisions of Kampala Capital City, Uganda. Private health Centre IIs, IIIs and clinics in the selected divisions where the TB focal person had not worked for at least six months since such a period would not give them sufficient time to know enough about barriers and facilitators of TB notification at the facility. TB focal persons are expected to be most involved in TB diagnosis and case notification at the selected facility.

### Study design

This was a cross-sectional study. We employed a mixed methods approach in which quantitative and qualitative data were collected and analyzed concurrently to gain a better understanding of the identified factors that act as barriers and facilitators of TB case notification. We also sought to capture qualitative information explaining some nuances observed at facility level as well as to obtain a deeper understanding of facilitators and barriers to TB notification at the facilities with high or low TB case notification rates.

### Sample size

Using the sample size estimation formula below by Kish Leslie [19], the sample size was calculated using a 95% confidence interval and 5% precision, and the expected prevalence of TB notification by private facilities in Uganda which is estimated to be 21% based on unpublished data from the NTLP. The resulting sample size was adjusted for a finite population and non-response rate of 10% catered for to obtain our final sample size of 238 private facilities.

### Sampling procedure

**Fig 1:**
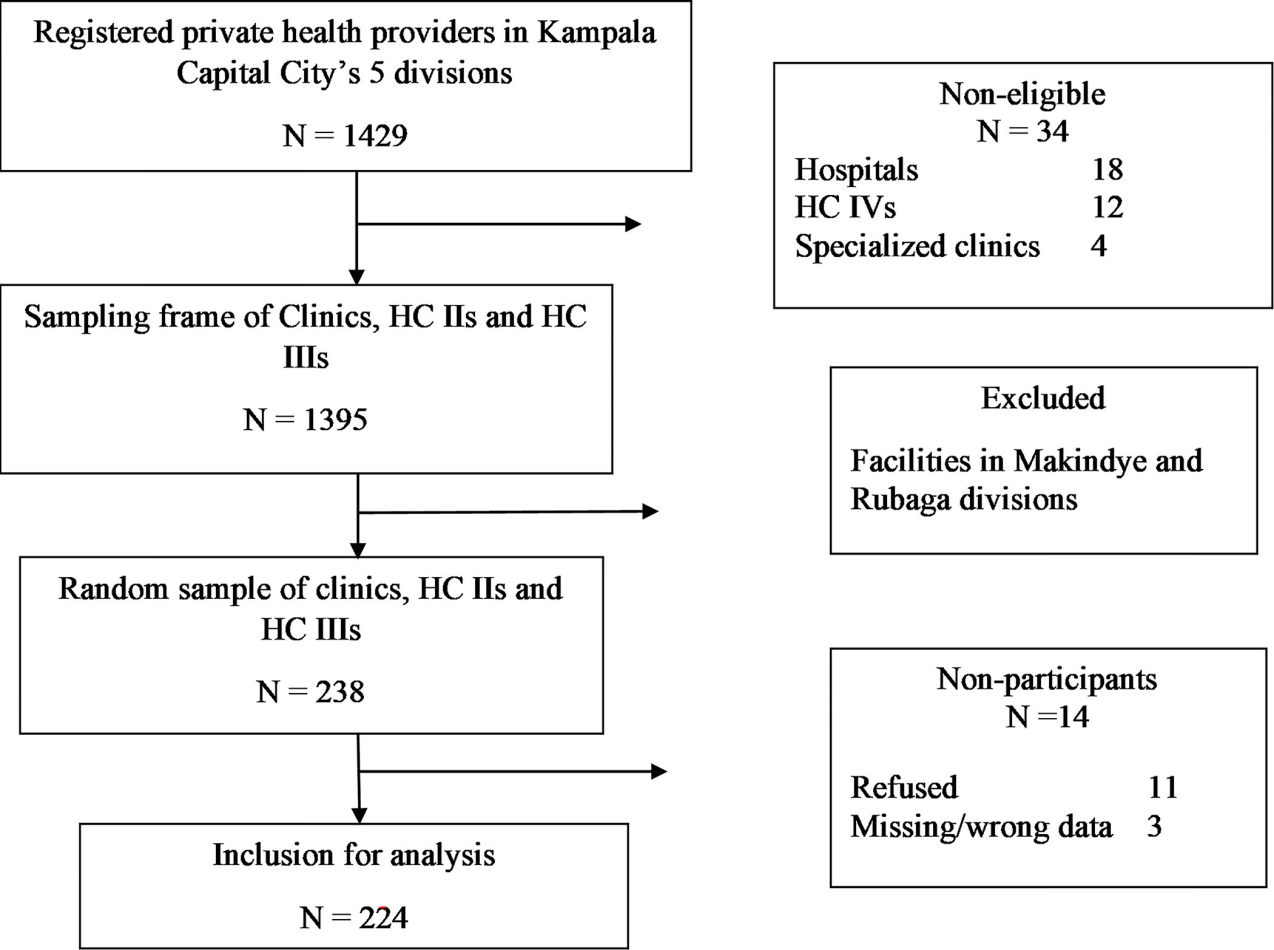
Sample selection flowchart.

Out of the five (5) administrative divisions of Kampala, we purposively selected three (3)—Central, Nakawa and Kawempe—because they are the most populated in Kampala and have higher poorer populations; conditions that pose the highest risk for TB transmission. Study units were sampled from the list of private facilities in DHIS2 which are expected to notify TB cases. Using the line list of DHIS2 registered private facilities in the study area for each of the three divisions, a number proportionate to the DHIS2 registered facilities in the division was randomly selected with the aid of computer-generated numbers **Fig 1**.

For the survey, at each facility we interviewed one (1) TB focal person at the facility and, in his/her absence, the substitute.

Participants for the KIIs and IDIs were selected using purposive sampling. The facilities for the In-depth interviews were selected according to high and low facility TB case notification: in each division, we selected one TB focal person from each of two (2) facilities with the highest case notification. Similarly, we had one TB focal person from each of the two facilities with the lowest TB case notification rates in each of the selected divisions. High and low notification was based on the facility’s TB screening numbers reported in DHIS2 between October and December 2021.

Two (2) KI interviews were conducted among two (2) NTLP staff including the Kampala region TB focal person and the PPM officer, and two (2) KI interviews were conducted among Kampala Capital City Authority TB focal persons from Central and Kawempe divisions. The Key Informants included individuals who are responsible for overseeing TB case management in private health facilities in Kampala Capital City and also have expert knowledge on and active involvement in support of TB case notification by PHPs.

### Study variables

The dependent variable in this study was notification of TB cases by private facilities. This was a binary variable defined by the number of PHPs that notify TB cases and the number of PHPs that do not notify TB cases. The number of PHPs that notify TB cases were obtained from the number of facilities of those recorded/registered in DHIS2 in the study area that notified TB cases. The number of PHPs that do not notify TB cases was obtained from the number of facilities in the study area that are recorded in DHIS2 but did not notify TB. This data was retrieved from the 033b Health Management Information System data in DHIS2 for the last quarter of 2021.

The independent variables included factors that influence the outcome (notification of TB cases) by private healthcare providers. These variables included: Level of knowledge of TB symptoms; degree of suspicion for TB; sufficient time for provider-patient interaction; willingness to notify a presumptive TB case; availability of guidelines for TB screening at the facility; availability of sufficient health care providers at the facility, level of turnover of health care workers at the facility, willingness of health care providers to engage in programmatic TB management, availability of diagnostic equipment and supplies, provision of TB case finding guidelines/tool kits by the NTLP, availability of specialized TB training for private health providers, TB stigma and health worker cadre.

### Data collection and management

Data was collected between March and June 2022. For the survey, we interviewed health workers in sampled PHFs using a structured questionnaire programmed in Open Data Kit (ODK) software platform.

We conducted in-depth interviews using in-depth interview guides and similarly used Key Informant interview guides to interview KIs. After making an appointment with the participants, an interview was held in which information on all objectives was gathered.

Five (5) research assistants were recruited and given rigorous training in collection of the quantitative data for this study, and they were supervised during the entire period of data collection. The filled tools were reviewed for completeness and any possible errors found in the data were corrected to enhance validity of the data. We also reviewed collected data to ensure that variables needed for analysis were captured in the data collection procedures.

A copy of the electronic data was saved as password protected and stored on an external storage device for safety and ethical purposes. The cleaned data was downloaded into Microsoft excel 2019 and imported into STATA 14 for statistical analysis.

### Data analysis

Based on DHIS2 data for the study facilities, we generated the outcome variable for notification in STATA and it was defined as the proportion of PHPs (private facilities) doing TB case notification: with “Yes” for those facilities that do (TB notification determined to be 15%) and “No” for those that do not do TB notification (85%).

Individual associations of each independent variable in the study with the outcome variable were tested using Fischer’s exact analysis in STATA. All independent variables whose p-value was less than 0.25, and other important variables with higher p-values were included in the model. We also tested for multicollinearity among the model variables and those whose correlation coefficient was more or equal to 0.4 were left out of the model for multivariable analysis. Since the prevalence of TB case notification among private facilities was 15%, we employed Modified Poisson for the multivariable analysis and, using parsimony, finally came up with a simple model with five (5) predictor variables. P-values for the adjusted Prevalence Ratios (PR) of the tested association of the predictor variables with TB case notification were obtained and used to determine the significance of the findings.

After interviews, audio recordings from the IDIs and KIIs were stored on an external storage device for safety and ethical purposes. We conducted thematic analysis, employing mainly a deductive process to analyze the data and using ATLAS.ti software to ease the analysis. Codes were pre-identified from the framework and then transcripts were reviewed as existing and emerging codes were identified and used to develop a codebook to guide the analysis in ATLAS.ti. The codes were arranged and merged to develop themes and sub-themes relating to common notification practices among the PHPs as well as the facilitators and barriers to TB case notification.

#### Ethical considerations

The study was approved by the Makerere University School of Public Health Higher Degrees Research and Ethics Committee. Permission to carry out this study in the three (3) divisions of Kampala was sought from the Kampala Capital City Authority, that is, KCCA Directorate for Public Health Services and Environment.

Written informed consent was obtained from the study participants prior to data collection. To obtain consent, each participant was given the consent form to read, allowed to ask any desired questions, then they signed the form if they agreed to participate. Indirect identifiers were used for all study participants.

## Results

We visited 238 private facilities from which we managed to interview a total of 224 health workers (6% non-response rate. The majority 39.3%(88) were from facilities in Nakawa division, 55.4% (124) were male and the mean age of facility health workers was 32.6 years (SD = 8.6) **Table 1**.

**Table 1:** Characteristics of facilities and respondents.

Factor	n	(%)
**Location of facilities by division**		
Central	56	25%
Kawempe	80	36%
Nakawa	88	39%
**Health Facility level of operation**ª		
Clinic	23	10%
Health centre II	195	87%
Health Centre III	6	3%
**Health Worker cadre**		
Doctor	39	17.4
Nurse	90	40.2
Clinical officer	55	24.6
Lab technician	16	7.1
Other*	24	10.7
**Number of years worked at the facility**		
Less than 1	31	13.84
1–4	116	51.79
5–9	48	21.43
10 and above	29	12.95
**Health worker trained in TB diagnosis**		
Yes	64	28.6
No	160	71.4
**Facility linked to notify TB cases through reporting tools**		
Yes	143	63.8
No/I don’t know	81	36.2
**Facility has guidelines for screening for TB**		
Yes	89	39.7
No	135	60.3

*Includes Midwife, counsellor, radiographer and staff without professional health training

ªEstimates based on facility classification in DHIS2

In the survey, we observed that, while majority of the health workers (58%) reported not to have notified a suspected TB case before, 46%(103) reported that their facility did regular TB notification using national reporting tools particularly with HMIS tools (TB/OPD registers) 40.2%(90). Only very few 1.8%(4) health workers reported use of the new electronic Case Based Surveillance System in their facility. 71%(82) of the participants reported that the other health workers in the facility had not received TB training because they had not been offered the opportunity by government (NTLP) or an Implementing Partner, and only 6%(7) said that they were not interested in such training. In the survey, we also observed that among many reasons for the facility not having ever notified a TB case, only 2%(3) reported the patient’s disagreement as a reason for not notifying.

We found strong evidence indicating that the prevalence of TB case notification was significantly lower for facilities in Kawempe (PR 0.16; 95%CI 0.05,0.47) and Nakawa (PR 0.39, 95%CI 0.21,0.73) than in Central division **Table 2**.

**Table 2:** Regression findings for facilitators and barriers to TB case notification.

Factor	TB case notification		Unadjusted PR (95%CI)	Adjusted PR (95%CI)
	**Yes n (%)**	**No n (%)**		
**Division**				
Central	18 (32.1)	38 (67.9)	1.0	
Kawempe	4 (5.0)	76 (95.0)	0.16 (0.06–0.44)***	0.16(0.06–0.47)**
Nakawa	11 (12.5)	77 (87.5)	0.39 (0.20–0.76)**	0.39(0.21–0.73)**
**Receive suspected TB cases**				
Yes	23 (14.1)	140 (85.9)	1.0	1.0
No	10 (16.4)	51 (83.6)	1.16 (0.59–2.30)	1.67(0.86–3.23)
**Guidelines for screening TB**				
Yes	19 (21.4)	70 (78.7)	1.0	1.0
No	14 (10.4)	121 (89.6)	0.49 (0.26–0.92)*	0.50(0.25–0.97)*
**Trained in TB diagnosis**				
Yes	17 (29.3)	41 (70.7)	1.0	1.0
No	12 (9.8)	110 (90.2)	0.34 (0.17–0.66)**	0.60(0.29–1.27)
I don’t know	4 (9.1)	40 (90.9)	0.31 (0.11–0.86)*	0.35(0.13–0.93)*

* p<0.05 ** p<0.01 *** p<0.001

Prevalence of TB case notification was lower (PR 0.50; 95%CI 0.25,0.97) among facilities that had no guide for TB screening and diagnosis compared to those that had a guide for TB screening.

We observed that the prevalence of TB case notification was lower among those facilities where training of other health workers at the facility in TB diagnosis was unknown (PR 0.35; 95%CI 0.13,0.93).

Regression analysis did not show prevalence of TB notification to increase with: the facility’s linkage to a public facility for TB notification; receiving suspected TB patients at the facility, nor having reporting tools.

We interviewed 14 Key informants from among the National TB program staff, KCCA Division TB focal persons and facility TB focal persons **Table 3**.

**Table 3:** Characteristics of Key informant interview and in-depth interview participants.

		Key Informants (n)	In-Depth Interview Participants (n)
**Division level**	Kawempe	1	4
	Central	1	4
	Nakawa	0	2
**Central level**	NTLP	2	0
**Facility level**	TBFP		8
	Other health worker		2

In the IDIs and KIIs, we identified three (3) main themes: Technical capacity, logistics and resources for TB case notification and nature of Tuberculosis **Table 4**.

**Table 4:** Summary of themes and sub-themes.

Facilitators and barriers to Tuberculosis case notification
Technical capacity • Regular supervision and engagements from NTLP • Incompleteness of TB tools and inconsistency in TB reporting • High staff turnover affecting the availability of staff with sufficient experience for TB case finding • Need for TB training through facility CMEs
Logistics and resources for TB case notification • Lack of financial support to conduct TB case finding such as screening activities in the community • Provision of TB tools, guidelines and IEC materials • Provision of TB diagnostic facilities • Lack of airtime to maintain to report/refer cases
Nature of the disease • TB stigma among patients and health workers • Patient confidentiality concerns

### Technical capacity

Key informants indicated that they lacked sufficient capacity to notify TB cases. They mentioned that efforts to build capacity of TB notification by the NTLP encourage them to get involved in programmatic TB management and actively do TB case notification. However, they noted that Support is largely target public facilities and the private are left out.

*“I have been here for almost 2 years. I have not had any team from either Ministry of health to talk about TB or to tell me anything about TB, to try to do anything about TB, nothing. And surprisingly, I’m here 5 days (a week) so I [would] know*.” *(IDI-10)*

A key informant at the national level acknowledged the lack of capacity building for PHF (Private Health Facility) providers, seeing it as a significant obstacle to improving TB case notification. They indicated that the resources allocated for capacity building are only for public facilities, as per the government’s plans. Additionally, they noted that the tools used for case notification are frequently updated, requiring ongoing capacity building to maintain effective notification.

*“[We need to] orient them regularly on the new developments because we change tools all the time.… As we go, as a district, probably to supervise public DTUs, we should be able to supervise the private*.” *(KI -2)*

Many health workers reported that missing data or gaps in the tools were a common occurrence in their facilities which sometimes was a result of insufficient knowledge on how to fill the tools, shortage of human staff and lack of time.

*“We need to be inducted in these books. So, you find people will miss some [information]…create gaps when we are filling in those tables. So, when the supervisors come, you will find some missing information; that was actually the biggest challenge. And there was no proper person responsible for that reporting—who follows those books closely*.” *(IDI-6)*

Similar to the above was the inconsistency in reporting as expressed below:

*“The challenge that we get, usually, (is) the reporting; the report is really big… sometimes they miss out many things, sometimes they don’t report at all*” *(KI-1)*

The health workers concurred on the importance of incorporating TB in the facility CMEs since many health workers lack sufficient knowledge on diagnosis and reporting of TB. The Key Informants and private health workers also proposed health education among cases, their families and the communities to increase TB case finding from the community.

### Logistics and resources for TB case notification

The health workers emphasized that the provision of funding for the case finding activities of community health workers helps them to be able to do TB case notification. Some reported that this support was previously provided by IPs but it had been withdrawn, and it was difficult for the facility health workers to go to the communities to do contact tracing or screen for TB among the people.

*“Ideally [for] private clinics, one of the things I have seen, [is] if there could be a way that they can be motivated… A motivation can be airtime; because some of them can [say] like, ‘I wanted to call you but I had not airtime. We are lacking this and that*…’ *And remember that person is telling you when you are going to compile a report, meaning that we are not going to get a correct report.” (KI -3)*

Another health worker also referred to the challenge the lack of funding for contact tracing saying:

*“A client comes in, [and] we just ask them, “how many people do you stay with? Is there anybody coughing?” (Some) come, others don’t come so we miss out them. That’s the challenge also to us because we don’t have facilitation for contact tracing and community mobilization,*…” *(IDI-2)*

Some private health workers also identified the shortage of staff to carryout notification (screening, data recording) and community case finding activities as a barrier to TB notification. Furthermore, unlike public facilities, private facilities were found to have a high staff turnover which results in a shortage of TB trained staff requiring regular training and CMEs.

*“There is also another challenge. We keep on changing these staff. We don’t have a fixed person, that is, the person [in-charge of] TB screening. So, if you inducted one person, the next month the boss may bring another person who doesn’t even know how to notify TB, [or to] use registers*.” *(IDI-6)*

Some health workers further reported the need for the government to provide the reporting tools, guidelines and IEC materials. In addition to this, they emphasized the challenge of lacking TB diagnostic equipment such as microscopes and especially GeneXpert.

*“I think if we can get our own GeneXpert machine it could be better. because we can have many samples. ZN also takes long due to few staff we have in the lab. And even the X-ray, the money for X-ray some people cannot afford to go for X-ray and even scan. But if we had some funding like for x-ray or scan, maybe the notification would be more. We shall be having more cases*.” *(IDI-5)*

Interestingly, the health workers also proposed digitalization of TB reporting systems as a way to improve TB case finding. This is because recording of TB data was felt to be tedious for the health workers and required employment of extra personnel, an extra expense for the facility.

Health workers noted that sometimes delays in Turn-Around-Time for GeneXpert test results led in Loss to Follow Up because the case had eventually moved from the area or even death because the case had approached the facility in a critical state. The shortage of time in some facilities also limited the ability to conduct screening appropriately on all people.

#### Nature of the disease

It was also generally noted that in many of the facilities, especially HIV clinics, people fear to be associated with TB—given the knowledge that it is a highly infectious disease—so they do not disclose correct history during screening process.

*“…only that some patients hide. They don’t want to show that they have TB. You can ask: ‘Do you have a cough?’ ‘Mmm… No.’ ‘As if I have heard you coughing.” ‘Yes. Anyway, it is starting, it is starting*.’” *(IDI-1)*

Another challenge regarding the nature of TB was its association with the poor/underprivileged persons which makes one deny the diagnosis. Some patient may even of refusing to go to a referred public facility, and yet the private facility does not have the diagnostic equipment to confirm TB.

*“Some patients […] don’t want to be notified because (their) details have to be taken: the names, the age… Actually, stigmatizing somebody that ‘You have TB’… somebody can even sue you. So, in private it is a bit challenging; people don’t want to be stigmatized …, especially the corporates who say, ‘Doctor, how can I get TB?’ So, it also becomes a bit hard to notify such a patient in the register; (to say) that [*…*], a minister has TB.”* (IDI-6)

## Discussion

In this study we found that TB case notification among Private Health Providers was mainly supported by the provision of technical support by the NTLP and the government implementing partners such as USAID, while a key barrier to TB case notification was TB stigma.

Inadequate training support to PHPs was found to be a major barrier to TB case notification. While many health partners together with National TB programs have trained and supported groups of health workers, more often emphasis is laid on government health facilities while few resources are set aside for PHPs. High health worker turnover at private facilities [20] further strains efforts to sustain TB PPM collaboration efforts in the area of training and mentorship.

Similar to other studies [21], our findings show that provision of support like regular supervision and engagement activities is a key facilitator of TB case notification in Kampala. For many PHPs, these activities are an indicator of the government’s acknowledgement of their presence [22] and committed support them. These activities also provide avenues for the PHPs to voice their challenges and what they may be lacking to do TB case notification at their facilities [23], especially when they are not certain about who to approach.

It is also clear from the findings that many PHPs in Kampala have a great need for capacity building and training in TB case finding and reporting. These engagements discussed above could provide avenues to identify the training support needs of PHPs as well as the means through which this support can be provided to the facilities.

Availability of motivators like funding/financial support for TB case finding activities [24] and TB training have been observed to facilitate TB notification. Training is generally appreciated as the health workers learn about TB notification through such activities [25–27].

The desire of PHPs for increased accessibility to diagnostic facilities like GeneXpert has also been observed in other studies [28]. In most cases, private facilities cannot afford to have GeneXpert machines and for many, they cannot even perform microscopy, forcing them to refer cases to public facilities and thus, they fail to diagnose drug-resistant TB.

Providing information on TB through IEC materials and other means of health education addresses barriers like TB stigma and can improve patient communication during triage as well as community case finding activities. Other studies on facilitators of TB notification show that health education and communication could improve TB case notification [29].

The evidence found in this study for lower notification in some divisions suggests the possible existence of certain local area factors that act as barriers to TB notification including training and support through Implementing Partners from division health care authorities. Higher notification rates from one division may have been fostered by TB screening campaigns that had been carried out around Kampala business center [30] shortly before this study was conducted. It may, therefore, be helpful therefore to conduct similar case finding activities in all other divisions.

As in many similar studies [23, 24, 31–34], we observed that both patient and health worker stigma was a main barrier to TB case notification among PHPs. This finding suggests a limited understanding of TB and a gap in TB health education among patients, the communities and even the health workers at the facilities.

The lack of guidelines as a common barrier to TB case notification among PHPs may limit thoroughness in screening, diagnosis and notification of TB [26, 35] as they may not know and utilize other avenues provided in the guidelines for what they may lack to improve TB case finding.

Incompleteness of tools and inconsistencies in the reporting process of some of the private facilities was reported along with changes in the tools and reporting systems that have occurred in the last few years. This finding is similar to one from Ethiopia [20] where poor recording and reporting culture due to health worker negligence was found to hinder TB case detection. These factors can be mitigated through regular supervision and engagements. In other studies, some private health workers reported that the changes in the notification system [36], present a challenge to doing TB notification. However, it was also noted that resources for support supervision are limited since PPM is not a priority for many international and local health partners. Efforts need to be made to ensure political commitment and organizational support for PPM engagement.

## Conclusions

While mandatory TB case notification is a policy in Uganda, this study highlights that many of the PHPs may not be up to date in referral and reporting processes and low TB case notification remains a significant challenge in Kampala. Private health facilities are not against the mandatory TB notification policy but they lack necessary diagnostic equipment and/or support to do contact tracing for their patients which hinders or slows down the process of TB case notification. Furthermore, we observed that many of the identified barriers in screening, diagnosis and reporting of PHFs can be mitigated with support supervision, training and engagement activities through which PHPs can be provided with up-to-date knowledge and guidance on TB notification.

We recommend that the NTLP allocates more resources towards PHP supervision and engagement; regular private facility Tuberculosis-related Continuous Medical Education sessions (CMEs) be leveraged upon and encouraged among PHPs as the NTLP works towards increasing supervision, mentorship and training for them. The training of private health workers by the NTLP and regional Implementing Partners can be designed to target nurses and clinical officers, especially in TB hotspots where TB case numbers are likely to be high and TB yield could be higher. PHPs with higher TB case notification numbers reported in DHIS2 can be targeted for case finding support and private facilities that do HIV care should be equipped with extra support for case notification activities such as training and monetary incentives for community health workers who identify suspected cases and link TB contacts from the community.

### Limitations and future study

In this study, only Ministry of Health accredited facilities which report in DHIS2 were studied, other studies may be conducted among unaccredited facilities which also need to notify TB cases. In the survey, the observation method was not employed thus limiting the possibility of getting data on the presence or absence of equipment and infrastructure that can act as facilitators or barriers to TB case notification. Data collected on TB screening, diagnosis and reporting practices was self-reported which may have given rise to some reporting bias.

## Supporting information

S1 File Data collection tools. (ZIP)

S1 Dataset (XLSX)
