# Supplementary material for: Facilitators and barriers to Tuberculosis case notification among private health facilities in Kampala Capital City, Uganda
Source: PLoS One. 2024 Dec 19;19(12):e0315402. doi: 10.1371/journal.pone.0315402 (PMC11658470; doi:10.1371/journal.pone.0315402)
Supplement: S1 File — (ZIP) [file pone.0315402.s001.zip › Key Informant Interview guide.pdf]

APPENDIX D: KEY INFORMANT INTERVIEW GUIDE

Adapted from (Ayakaka et al., 2017)

Division:		Date:	
Type of KII:		Health facility:	
Name of interviewer:			
Start:		End:	

After a brief introduction to the participant regarding the purpose of the interview, the interviewer will take informed written consent for the interview. Written informed consent will also be requested for audio recording.

> *Now, I would like to ask you a few questions. Before I start let me stress that there are no right or wrong answers, no desirable or undesirable answers. Please feel free to say what you really think or feel.*
>
> *I am going to ask you some questions about Tuberculosis notification. You do not have to answer these questions if you do not want to. As a reminder, your responses will be anonymized, meaning that your name will not be attached, and I will not share your answers with others.*

1. Would you like to tell me a bit about yourself, who you are and what you do?
2. Describe the process of Tuberculosis case notification in private facilities in Kampala?
   Probes: The process, policy requirements for reporting (Scope of reporting, timing, who should report). Extent to which private clinics are supportive of programmatic TB case notification.
3. Share with me your experience and any engagement with Private Health Providers in Kampala on TB case notification?
   Probes: Willingness to report, present opportunities, challenges encountered, provision of incentives for reporting.

4. What kind of support do you provide to private clinics in Kampala to improve TB case notification?
5. Who are the major partners supporting Private Health Providers in Kampala in TB case notification?

   Probe: Nature of support by each partner.
6. What do you think can be done to improve TB case notification from private clinics in Kampala?

   Probes: system/policy level recommendations, facility accreditation requirements. Facility level recommendations, proposals for incentives that can be given.
7. As we conclude. Share any final thoughts relating to TB case notification that we may not have talked about.
