# Supplementary material for: Facilitators and barriers to Tuberculosis case notification among private health facilities in Kampala Capital City, Uganda
Source: PLoS One. 2024 Dec 19;19(12):e0315402. doi: 10.1371/journal.pone.0315402 (PMC11658470; doi:10.1371/journal.pone.0315402)
Supplement: S1 File — (ZIP) [file pone.0315402.s001.zip › Questionnaire.pdf]

APPENDIX B: PRIVATE HEALTH PROVIDER QUESTIONNAIRE

Interviewer: Date:

Section A: Health Care Provider Socio-demographics

1. Individual Code Number: ……………………
2. Division: ❑Central ❑Kawempe ❑Nakawa
3. Sex: ❑Female ❑Male
4. Age in complete years:
5. What is your occupation?

❑Doctor ❑Nurse ❑Clinical Officer ❑Laboratory technician

❑ Other

6. What is your level of qualification?

❑Certificate ❑Diploma in health sciences ❑Undergraduate degree in health sciences ❑Master's degree in health sciences

Section B: Health Care Provider

7. How many years have you worked in this facility? (0 for less than a year)

………………………

8. Do you receive suspected TB patients at this facility?

❑Yes ❑No

9. What are the signs and symptoms of TB?

❑Cough for over 2 weeks

❑Night Fevers

❑Excessive weight loss

❑Night sweats

❑Poor weight gain in children

10. Do you know how to screen for a presumptive TB case?

❑Yes ❑No

11. If no, why?

☐Little experience; a recent graduate

☐No guidelines

☐Lack of diagnostic facilities

12. If yes, how confident?

☐Very confident ☐Confident ☐Not confident

Section C: Health Facility

13. Does your facility have guidelines for screening of TB (TB diagnostic algorithm)?

☐Yes ☐No

14. If yes. With what frequency do you follow the guidelines in identifying a presumptive TB case?

☐Always ☐Often ☐Sometimes ☐Never

15. If no, why?

☐The guidelines are not very accessible (not fixed in a visible place)

☐They are out of date

☐I forget to use them

16. Would you say that you have enough time with patients to be able to screen for TB/ identify a presumptive TB case?

☐Strongly agree ☐Agree ☐Disagree ☐Strongly disagree

17. Does your facility have any channels for notifying TB cases to the Ministry?

☐Yes ☐No ☐Don't know

18. Does your clinic do regular TB case notification using national reporting tools?

☐Yes ☐No ☐Don't know

19. If yes, what tools do you use? (Tick all that apply)

☐TB register ☐SMS ☐DHIS2 ☐eCBSS

20. Have you notified a presumptive TB patient before?

☐Yes ☐No

21. If no, why?

☐I don't know

☐I'm not certain how

☐I fear to make a wrong diagnosis.

☐My presumptive TB patient did not agree to that.

☐I don't want to be followed up by government.

☐Filling TB registers and forms takes a lot of time.

☐I have a lot of work to do.

☐Other

22. Would you notify presumptive TB cases if you were given incentives?

☐Yes ☐No

23. Is your facility linked to any public facility for presumptive TB case referrals?

☐Yes ☐No

Section D: Health System

24. Have you obtained further training in TB diagnosis?

☐Yes ☐No

25. If yes, who organized this training?

☐My employer – privately

☐NTLP

☐Implementing Partner USAID (Defeat-TB), Uganda Stop TB Partnership (USTP)

26. Has any other health worker from your facility received any training in TB diagnosis and notification?

☐Yes ☐No

27. If no, why?

☐They are not interested

☐No one has been offered an opportunity (government or Implementing partner).

☐I don't know
