# Supplementary material for: Facilitators and barriers to Tuberculosis case notification among private health facilities in Kampala Capital City, Uganda
Source: PLoS One. 2024 Dec 19;19(12):e0315402. doi: 10.1371/journal.pone.0315402 (PMC11658470; doi:10.1371/journal.pone.0315402)
Supplement: S1 File — (ZIP) [file pone.0315402.s001.zip › In-depth interview guide.pdf]

APPENDIX C: IN-DEPTH INTERVIEW GUIDE

Adapted from (Ayakaka et al., 2017)

Division:		Date:	
Type of IDI:<br>(Doctor, nurse, clinic officer, lab technician)		Health facility:	
Name of interviewer:			
Start:		End:	

After a brief introduction to the participant regarding the purpose of the interview, the interviewer will take informed written consent for the interview. Written informed consent will also be requested for audio recording.

Now, I would like to ask you a few questions. Before I start let me stress that there are no right or wrong answers, no desirable or undesirable answers. Please feel free to say what you really think or feel.
I am going to ask you some questions about Tuberculosis notification. You do not have to answer these questions if you do not want to. As a reminder, your responses will be anonymized, meaning that your name will not be attached, and I will not share your answers with others.

1. Would you like to tell me a bit about yourself, who you are and what you do in life?
2. Tell me what you know with regard to Tuberculosis notification?
3. How would you go about notifying a presumptive/suspected case of TB from your clinic?
   Probes: Activities and offices/cadres involved. Timing, flexibility in reporting the data, etc.
4. The Ministry of Health requires that all TB cases are notified. Describe ways in which your clinic is supportive of TB case notification?

5. What processes do you go through in referring presumptive TB patients to other facilities like a public facility for TB diagnosis?

   Probes: Number of referrals made in the previous reporting period (year).

6. What challenges do you encounter in TB case notification for this facility?

7. Share with us your experience in notifying suspected TB cases to the National TB and Leprosy Control Programme through the HMIS/DHIS2?

8. What do you think can be done to improve TB case notification in this facility?

   Probes: Incentives that should be given to the facility and health workers to boost TB case notification.

9. As we conclude, share with us anything else about TB case notification at this facility that we may not have talked about?
